# Los inicios de la vacunación contra la viruela en Navarra: La actividad del médico Manuel Gil y Albéniz (1801-1836)

**DOI:** 10.23938/ASSN.1167

**Published:** 2026-07-21

**Authors:** Ramón Santonja Alarcón, Carlos González Guitián, Jose Tuells

**Affiliations:** 1 Universidad CEU Cardenal Herrera Escuela Internacional de Doctorado-CEINDO Programa de Doctorado en Humanidades para el Mundo Contemporáneo Elche Alicante España; 2 Instituto «José Cornide» de Estudios Coruñeses La Coruña España; 3 Universidad CEU Cardenal Herrera Facultad de Ciencias de la Salud Elche Alicante España; 4 France, Amériques, Espagne, Sociétés, Pouvoirs, Acteurs (FRAMESPA) Unidad Mixta de Investigación del Centre National de la Recherche Scientifique (CNRS) y de la Université Toulouse - Jean Jaurès Toulouse Francia

**Keywords:** Vacunación, Viruela, Historia de la Medicina, Salud pública, Navarra, Manuel Gil y Albéniz, Vaccination, Smallpox, History of Medicine, Public Health, Navarre, Manuel Gil y Albéniz

## Abstract

**Fundamento:**

La introducción de la vacunación contra la viruela a comienzos del siglo XIX supuso uno de los principales avances en la historia de la salud pública en España. Aunque su difusión inicial ha sido estudiada principalmente desde las iniciativas institucionales, se conoce menos el papel de los médicos locales en su implantación.

**Material y métodos:**

Se realizó un estudio histórico-documental basado en el análisis de fuentes primarias (archivos, prensa histórica y memorias médicas) y secundarias, centrado en la trayectoria del médico navarro Manuel Gil y Albéniz (1767-1849), titular de Cascante (Navarra).

**Resultados:**

Gil y Albéniz inició la vacunación contra la viruela en Cascante en 1801, en una fase temprana de su introducción en España. Desarrolló una intensa actividad como vacunador, alcanzando centenares de vacunaciones y contribuyendo a la difusión del material vacunal en Navarra. Sus escritos documentan métodos de conservación y transmisión de la vacuna, así como estrategias para mantener la vacunación durante la Guerra de la Independencia. Participó en redes científicas nacionales como socio corresponsal de la Real Academia de Medicina de Barcelona y de la Sociedad Médico-Quirúrgica de Cádiz. En 1820 publicó la *Colección de memorias médicas*, que recoge sus principales observaciones.

**Conclusiones:**

Manuel Gil y Albéniz puede considerarse uno de los introductores tempranos de la vacunación en Navarra. Su trayectoria ilustra el papel de los médicos locales en la difusión de la vacuna y en la consolidación inicial de las prácticas de salud pública en la España del siglo XIX.

## Abstract (en)

**Background:**

The introduction of smallpox vaccination in the early nineteenth century marked a major milestone in the history of public health in Spain. Although its early dissemination has been mainly studied from an institutional perspective, the contribution of local physicians to its implementation remains less well understood.

**Methods:**

Historical study based on documentary sources conducted using primary sources, including archival records, historical newspapers, and medical memoirs, together with relevant secondary literature. The study focuses on the career of the Navarrese physician Manuel Gil y Albéniz (1767-1849), who practised in the town of Cascante (Navarre).

**Results:**

Gil y Albéniz introduced smallpox vaccination in Cascante in 1801, during the early stage of its adoption in Spain. He carried out hundreds of vaccinations and played an important role in disseminating vaccine material throughout Navarre. His writings describe methods for preserving and transmitting vaccine material and document strategies that enabled vaccination to continue during the Peninsular War. He also participated in scientific networks as a corresponding member of the Royal Academy of Medicine of Barcelona (1803) and the Medical-Surgical Society of Cádiz (1820). His *Colección de memorias médicas* (1820) brings together his principal clinical observations.

**Conclusions:**

Manuel Gil y Albéniz was among the earliest physicians to introduce smallpox vaccination in Navarre. His career highlights the important contribution of local physicians in the dissemination of vaccination and the early development of public health in nineteenth-century Spain.

## INTRODUCCIÓN

La viruela fue una de las enfermedades infecciosas más devastadoras en Europa, con tasas de mortalidad que afectaban especialmente a la población infantil. Durante el siglo XVIII se difundió la práctica de la variolización, consistente en la inoculación deliberada de material procedente de enfermos con el objetivo de provocar en el receptor una forma leve de la enfermedad y, posteriormente, generar inmunidad. Sin embargo, los riesgos asociados limitaron la aplicación de este procedimiento. El descubrimiento de la vacunación por Edward Jenner en 1796 supuso una alternativa a la variolización más segura y eficaz, que comenzó a difundirse rápidamente por Europa y fue introducida en España a comienzos del siglo XIX^1^^,^^2^.

La historiografía ha prestado especial atención a las iniciativas impulsadas desde la administración central, entre las que destaca la expedición filantrópica de la vacuna dirigida por Francisco Javier Balmis (1803-1813), considerada un hito en la difusión internacional de la vacunación^3^. Sin embargo, la implantación efectiva de la vacuna en el territorio español dependió de la interacción entre centros científicos, como la corte de Madrid, y la actuación de médicos locales que organizaron campañas de vacunación, transmitieron el material vacunal y difundieron su uso entre la población. En regiones como Navarra y Aragón, su distancia respecto a los principales centros académicos y administrativos hacía que la implantación de nuevas prácticas sanitarias recayera en la iniciativa individual de los médicos y cirujanos locales.

En este proceso desempeñaron un papel clave figuras como Ignacio María Ruiz de Luzuriaga, quien desde la Real Academia de Medicina de Madrid articuló redes de correspondencia y distribución del fluido vacunal a escala nacional, junto con redes profesionales y científicas formadas por academias, sociedades médicas y la comunicación entre facultativos^4^^,^^5^. Entre los médicos que participaron activamente en la difusión temprana de la vacuna destaca Manuel Gil y Albéniz (1767-1849), médico titular de la localidad navarra de Cascante, cuya actividad ha sido puntualmente mencionada por la historiografía, pero apenas analizada de forma sistemática. Aunque hasta ahora solo se disponía de referencias breves^6^^-^^10^, estudios recientes han comenzado a explorar su relación con las academias de Cádiz y Barcelona, abriendo nuevas vías de investigación^11^^,^^12^.

El presente trabajo tiene como objetivo analizar la introducción y difusión de la vacunación contra la viruela en Navarra a través de la trayectoria profesional de Manuel Gil y Albéniz, integrando por primera vez documentación de archivo dispersa en múltiples instituciones para reconstruir su labor como vacunador y su participación en las redes científicas de la época.

## MATERIAL Y MÉTODOS

Se realizó un estudio histórico-documental basado en el análisis de fuentes primarias y secundarias relativas a la introducción y difusión de la vacunación contra la viruela en Navarra durante las primeras décadas del siglo XIX, centrado en la figura de Manuel Gil y Albéniz (1767-1849), con el objetivo de analizar su papel en la implantación y propagación de la práctica vacunal en el ámbito local y su relación con las redes médicas de la época.

Las fuentes primarias proceden de archivos militares, universitarios, académicos y locales, incluyendo documentación personal, correspondencia científica, memorias médicas y registros civiles y parroquiales. Asimismo, se analizaron publicaciones del propio autor y prensa histórica del periodo. Las fuentes secundarias incluyen trabajos historiográficos sobre la introducción de la vacuna en España y estudios recientes sobre Gil y Albéniz.

El análisis se realizó mediante métodos de investigación histórica basados en la contextualización y comparación de testimonios. Estos documentos permiten reconstruir tanto su actividad clínica como su participación en redes profesionales dedicadas a la difusión de la vacunación.

## RESULTADOS

Los principales hitos biográficos y profesionales de Gil y Albéniz se resumen en la Tabla 3, al final de esta sección.

### Formación y primeros años de ejercicio profesional

Manuel Gil y Albéniz nació en Cascante (Navarra) el 9 de junio de 1767^13^ en el seno de una familia que ocupaba una posición relevante en la localidad, ya que su padre ejerció el cargo de regidor municipal^14^.

Este entorno favoreció su acceso a una formación académica en el ámbito médico. Realizó sus estudios en la Universidad de Zaragoza, donde obtuvo el grado de bachiller en Medicina en 1789, completando posteriormente su formación práctica. Tras ser examinado por el Colegio Médico de Pamplona, ejerció en distintas localidades navarras antes de establecerse definitivamente en Cascante en 1797 como médico titular^15^. Contrajo matrimonio con Manuela Montorio de Malo (1768-1834) el 3 de marzo de 1794, sin descendencia^16^.

### La práctica médica y el paso de la inoculación a la vacunación

Gil y Albéniz señaló en sus escritos que “*hallábame inoculando la viruela natural cuando la vacuna llegó a nuestro suelo*”. Según sus testimonios, la inoculación de la viruela natural ya se realizaba en Pamplona cuarenta años antes de la introducción de la vacuna^17^.

La aparición de la vacunación jenneriana a finales del siglo XVIII supuso una alternativa más segura a la inoculación, lo que llevó a muchos médicos a abandonar progresivamente esta técnica y adoptar el nuevo método preventivo. Gil y Albéniz se incorporó tempranamente a la práctica de la vacunación contra la viruela, convirtiéndose en uno de los primeros médicos que introdujeron esta técnica en Navarra^18^. Sus memorias médicas reflejan una práctica basada en la observación clínica y el intercambio de conocimiento sobre epidemias, vacunación y métodos terapéuticos, que muestran su voluntad de contribuir al conocimiento médico de su tiempo.

### Introducción de la vacunación en Cascante (1801)

La vacuna contra la viruela comenzó a difundirse por Europa en los primeros años del siglo XIX^2^. En España su introducción se produjo en torno a 1800, impulsada tanto por iniciativas individuales de médicos y cirujanos como por programas institucionales.

La vacuna llegó a Cascante en noviembre de 1801^19^, constituyendo uno de los primeros focos documentados de vacunación en Navarra^16^. El material vacunal fue trasladado desde la localidad aragonesa de Magallón por un vecino destacado, Francisco Sánchez, quien había obtenido el denominado *germen vacuno*, que permitió iniciar la vacunación de los primeros niños de la población bajo la supervisión de Gil y Albéniz^20^.

En los meses siguientes la vacunación se extendió rápidamente entre la población local. En el parte que Gil y Albéniz remitió a la Gaceta de Madrid, fechado el 4 de mayo de 1802, contabilizaba más de ciento treinta vacunados^19^, cifra también recogida por el médico francés François-Emmanuel Fodéré ese mismo año en su obra sobre medicina legal^21^.

Gil y Albéniz mantenía relación con Vicente Martínez Monreal, a quien llamaba *mi condiscípulo*, quien en su *Tratado histórico práctico de la vacuna* (1802) señalaba: “don Manuel Gil, médico de la misma ciudad, la ha extendido en todo el vecindario [de Cascante]”^22^. La introducción en Cascante formó parte de una red de transmisión de la vacuna en Navarra; Martínez Monreal documentó que Pamplona, Tudela y Errazu fueron las primeras poblaciones navarras en introducir y propagar la vacuna, ya fuese con fluido transportado en cristales o de brazo a brazo^17^^,^^23^^,^^24^.

### Actividad vacunal y desarrollo de la práctica preventiva

Tras la introducción de la vacuna, Gil y Albéniz desarrolló una intensa actividad como vacunador. Entre noviembre de 1801 y noviembre de 1805, según la memoria *Historia de la vacuna en treinta años continuos* que remitió a la Real Academia de Medicina de Barcelona en 1831, había vacunado a 973 personas, entre niños y adultos de ambos sexos, con solo cinco casos de falsificación (ausencia de respuesta vacunal efectiva o ausencia de desarrollo de la lesión característica tras la inoculación) y dos de personas que padecieron la viruela natural a pesar de haber sido vacunadas^17^^,^^25^. En la memoria sobre la vacuna remitida a la Junta Superior de Sanidad el 10 de noviembre de 1814, Gil y Albéniz afirmaba que durante los primeros trece años de actividad vacunal había alcanzado aproximadamente la cifra de 7.000 vacunaciones^25^. Estas cifras, aunque deben interpretarse con cautela, reflejan la continuidad de su actividad y su implicación en la difusión de la vacuna en la región (Tabla 1).

**Tabla 1:** Actividad vacunal documentada de Manuel Gil y Albéniz

Fecha	Número de personas vacunadas	Fuente
1801, noviembre	Primer caso documentado	Archivo RAMC, 1831^17^
1802, mayo	>130 vacunadas	Gaceta de Madrid^24^
1802	>130 vacunadas	Fodéré^20^
1805	973	RAMC,1831^17^
1814	~7.000 vacunadas	*Colección de memorias médicas*^25^

RAMC: Real Academia Médica de Cádiz.

### Problemas técnicos y conservación del material vacunal

Durante los primeros años de aplicación de la vacuna surgieron diversas dificultades técnicas relacionadas con la conservación y transmisión del fluido vacunal. Uno de los métodos más habituales consistía en la transmisión directa *de brazo a brazo*, es decir, utilizando el material procedente de la pústula de una persona recientemente vacunada para inocular a otra; era el método más fiable, pero no siempre era posible.

La conservación del material vacunal en pequeños recipientes de vidrio o cristales permitía su transporte, pero presentaba limitaciones, especialmente en contextos bélicos como durante la Guerra de la Independencia (1808-1814). En sus escritos, Gil y Albéniz describió varios intentos fallidos de conservar la vacuna en cristales entre julio de 1808 y febrero de 1809, lo que provocó interrupciones temporales en la práctica vacunal. No fue hasta abril de 1809 cuando pudo retomarla parcialmente^17^.

Ante estas dificultades, el médico navarro propuso utilizar las postillas (costras secas formadas en el lugar de la vacunación) como medio para conservar y transportar el material vacunal, en lugar del método *brazo a brazo*. Según sus observaciones, este procedimiento facilitaba el transporte del material y permitía reactivar la vacunación cuando fuese necesario, siendo más eficaz que el almacenamiento en cristales, ya que “*la actividad del fluido vacuno cristalizado en la postilla para conservarlo y reproducirlo cuando se necesite y quiera*” era superior. Una sola postilla podía equivaler a varios recipientes de fluido vacunal y podía enviarse fácilmente por correo^17^^,^^22^. Este “*descubrimiento, obra de mi genio investigador”*, como él mismo lo calificó, le permitió difundir la vacuna mediante correspondencia^17^. Años más tarde, publicaciones como El Siglo Médico recordarían sus esfuerzos para conservar y propagar la vacuna durante la guerra, citándole *con justo elogio*^10^^,^^17^.

Al finalizar la guerra, publicó en agosto de 1814 un anuncio en la Gaceta de Madrid ofreciendo remitir vacuna gratuitamente a toda la península mediante postillas^24^. La respuesta fue notable: logró reactivar la vacunación en varios lugares, y envió material a 72 destinatarios, entre ellos 15 cirujanos, 12 médicos y 10 clérigos^25^. En su *Estado actual de la vacunación en España* (1815), incluyó un detallado listado de corresponsales que habían solicitado la vacuna, demostrando la amplitud de su red^17^.

### Redes profesionales y actividad científica

La actividad de Gil y Albéniz trascendió el ámbito local gracias a su participación en redes científicas nacionales. En 1802 inició contacto con la Real Academia de Medicina de Barcelona, remitiendo una memoria topográfica sobre Cascante y un análisis de las aguas de la fuente del Matador. Cumplidos los requisitos, el 5 de noviembre de 1803 fue admitido como socio corresponsal^18^.

Asimismo, mantuvo correspondencia con la Real Junta Superior Gubernativa de Medicina en Madrid, a la que remitió diversos informes sobre la práctica de la vacunación y sus observaciones clínicas. En noviembre de 1805 fue comisionado oficialmente por dicha Junta para observar, describir y propagar la vacuna por Real Orden^17^.

El 11 de noviembre de 1820 fue nombrado socio corresponsal de la Sociedad Médico-Quirúrgica de Cádiz tras enviar una topografía médica de Cascante que fue reseñada en el Periódico de dicha institución^25^. Mantuvo una relación continuada enviando memorias hasta 1828^26^^-^^29^.

Gil y Albéniz defendió con vehemencia una tesis audaz para su época: la capacidad de la vacuna de la viruela para prevenir no solo dicha enfermedad, sino también el sarampión y la escarlatina. Esta convicción, basada en su práctica clínica en Cascante, le llevó a sostener que la vacunación universal *depuraba* el organismo de otras afecciones cutáneas^25^.

Esta postura generó una intensa controversia pública en 1817, tras la publicación de sus tesis en la Crónica Científica y Literaria^30^. Sus afirmaciones fueron duramente criticadas por Jacinto Pazos de Vigo desde el Diario Mercantil de Cádiz, quien cuestionó la validez de generalizar una experiencia local y recordó que en Cádiz muchos niños vacunados habían contraído el sarampión ese mismo invierno^30^.

Gil y Albéniz respondió defendiendo la *verdad* de su experiencia frente a la *lógica* de su rival, pero Pazos mantuvo sus dudas en una contrarréplica final^30^. A pesar de la falta de base científica aceptada, Gil y Albéniz mantuvo su postura durante décadas, reafirmándola en su memoria de 1831 a la Academia de Barcelona^17^.

En 1816, su descripción de una epidemia ocurrida en Cascante en 1803-1804 fue premiada por la Real Academia de Medicina de Barcelona en el concurso de *Epidemias*, y recibió una medalla. Gil y Albéniz expresó su agradecimiento y envió otras contribuciones a lo largo de los años^31^^-^^33^.

En 1820 publicó su obra cumbre, la *Colección de memorias médicas*, que recopilaba la memoria premiada sobre epidemias, cinco memorias sobre vacunación presentadas a la Junta Superior, y un escrito sobre el uso de la quina en el tratamiento de calenturas^25^. La obra fue reseñada elogiosamente por Francisco Javier Laso en el Periódico de la Sociedad Médico-Quirúrgica de Cádiz^26^.

En sus últimos años, además de continuar con la práctica clínica y la elaboración de memorias, como la del cólera en 1836^31^, Gil y Albéniz comercializó un *elixir nervino* -preparado terapéutico empírico de la época sin eficacia validada según criterios científicos actuales- para tratar la alferecía, término histórico empleado para designar las convulsiones infantiles. Dio cuenta de este elixir a la Academia de Cádiz en 1828^29^ y lo promocionó en prensa, mostrando una faceta de médico emprendedor^34^.

La Tabla 2 resume las principales comunicaciones científicas enviadas a la Sociedad Médico-Quirúrgica de Cádiz y a otras instituciones, recogidas en su *Colección de memorias médicas*.

**Tabla 2:** Principales comunicaciones científicas de Manuel Gil y Albéniz

Fecha	Trabajo	Institución
1803	Descripción histórico-médica de la epidemia de 1803-1804^25^	Academia Médica de Barcelona
1807, 2 de diciembre	Parte sobre escarlatina en Cascante y vacunación^25^	Junta Superior de Medicina
1814, 10 de noviembre	Memoria sobre la vacunación^25^	Junta Superior de Medicina
1815, 10 de mayo	Estado actual de la vacunación en España y lista de corresponsales^25^	Junta Superior de Medicina
1816	Parte sobre vacunación y sus ventajas, con testimonios de diversos profesionales^25^	Junta Superior de Medicina
1820, 15 de julio	Apuntes sobre el modo de propagar la inoculación de la vacuna^25^	Sociedad Médico-Quirúrgica de Cádiz
1821, 1 de septiembre	Topográfico-médica de Cascante^22^	Sociedad Médico-Quirúrgica de Cádiz
1823, 26 de febrero	Observaciones sobre exantemas^11^	Sociedad Médico-Quirúrgica de Cádiz
1824, 1 de marzo	Parte de la vacunación en la época trágica constitucional^17^	Sociedad Médico-Quirúrgica de Cádiz
1824, 1 de mayo	Consideraciones sobre la vacuna^19^	Academia Médica de Barcelona
1828, 17 de junio	Sobre las virtudes del elixir nervino^28^	Sociedad Médico-Quirúrgica de Cádiz
1831, 7 de septiembre	Historia de la vacunación en treinta años^17^	Academia Médica de Barcelona
1832	Inspección de las fiebres epidémicas de Tarazona^32^	Academia Médica de Barcelona
1836	Memoria sobre el cólera en Cascante^31^	Academia Médica de Barcelona

### Reconocimiento, aspiraciones y últimos años

El 22 de julio de 1815 se le concedieron los honores, uso de uniforme y fuero de médico de número del ejército por sus méritos y servicios no especificados^15^.

En 1823, ante una grave epidemia de viruela en Andalucía que había agotado la vacuna en Cádiz, Sevilla y Gibraltar, Francisco Javier Laso recurrió a Gil recordando su fama y su ofrecimiento público para que enviase material vacunal^17^. Esta solicitud pudo impulsar a Gil a solicitar, a través del virrey de Navarra, una ampliación de su comisión de vacunas a las capitales de provincia y una pensión vitalicia. Sin embargo, esta petición fue denegada en diciembre de 1824 por el Rey, argumentando que las leyes de 1805 ya regulaban la materia y que no procedía gravar al erario^15^.

Sus aspiraciones de obtener la plaza de Protomedicato de Navarra tampoco se materializaron. En 1824 solicitó el apoyo de Palafox, llegando a ofrecer incluso un desembolso económico para facilitar las gestiones, pero sin éxito^35^. Residió en Pamplona entre 1825 y 1827 con la esperanza de obtener el cargo y encargarse de los niños de la Inclusa, pero hubo de regresar a Cascante sin haberlo conseguido^17^.

Políticamente, Gil se definió como *realista fino* en 1823^15^, lo que le llevó a ser investigado y finalmente *purificado* por la Junta de Pamplona en 1825^15^.

Gil, ya viudo y sin descendencia, otorgó varios testamentos (1837, 1843, 1845) en los que dejaba como herederos a sus sobrinos^37^. Falleció en Cascante el 22 de febrero de 1849, a los ochenta y un años de edad^38^.

**Tabla 3:** Principales hitos en la trayectoria de Manuel Gil y Albéniz

Año	Acontecimiento
1767	Nacimiento en Cascante
1789	Bachiller en Medicina por la Universidad de Zaragoza
1791	Médico titular de Cortes
1792	Aprobado en el Colegio Médico de Pamplona
1793	Médico del hospital de Elizondo (Guerra de la Convención)
1793	Médico titular en Mendigorría
1794	Matrimonio con Manuela Montorio de Malo
1797	Médico titular de Cascante
1798	Médico auxiliar de Cintruénigo
1801	Introducción de la vacunación en Cascante
1803	Socio corresponsal de la Academia Médica de Barcelona
1805	Comisionado por la Junta Gubernativa de Medicina para propagar la vacuna
1808-1814	Actividad durante la guerra de la Independencia; desarrollo del método de conservación mediante postillas
1814	Ofrece remitir vacuna gratuitamente a toda España
1815	Concesión de honores, uniforme y fuero de médico de número del Ejército
1817	Polémica pública con Jacinto Pazos de Vigo sobre la acción de la vacuna
1820	Publicación de *Colección de memorias médicas*
1820	Socio corresponsal de la Sociedad médico-quirúrgica de Cádiz
1823-1824	Propuesta de vacunación en las capitales de provincia
1825-1827	Estancia en Pamplona
1832	Inspección de epidemia en Tarazona
1836	Memoria sobre el cólera
1849	Fallecimiento en Cascante

## DISCUSIÓN

La introducción de la vacunación contra la viruela en España a comienzos del siglo XIX constituyó un proceso complejo en el que intervinieron tanto iniciativas institucionales como la actividad de médicos locales. La trayectoria de Manuel Gil y Albéniz constituye un ejemplo representativo del papel desempeñado por los médicos de ámbito local en la difusión temprana de la vacunación en España. La introducción de la vacuna en Cascante en 1801 la sitúa entre los primeros focos de implantación en Navarra. Además, el elevado número de vacunaciones documentadas durante los años siguientes refleja la consolidación progresiva de esta práctica preventiva en el ámbito local, documentando más de 7.000 vacunados en trece años^26^. No obstante, debe señalarse que una parte sustancial de la información cuantitativa (número de vacunaciones, continuidad de la práctica, cobertura) procede de escritos autobiográficos del propio Gil y Albéniz. Aunque se introducen cautelas interpretativas, es necesario explicitar el posible sesgo de autorrepresentación, frecuente en este tipo de memorias profesionales. Por ello, las cifras aportadas por el propio Gil deben interpretarse con cautela, como reflejo de su experiencia declarada más que como estadísticas exhaustivas.

Otro aspecto relevante es la participación de Gil y Albéniz en redes profesionales y científicas dedicadas a la circulación del conocimiento médico. Su correspondencia con la Real Junta Superior Gubernativa de Medicina, así como su relación con instituciones como la Real Academia de Medicina de Barcelona o la Sociedad Médico-Quirúrgica de Cádiz, evidencian la existencia de redes médicas para el intercambio de conocimiento y material vacunal sobre la aplicación de la vacuna, y su contribución a la creación de una red de intercambio de conocimiento y el envío gratuito de material vacunal a toda España desde 1814^27^^,^^33^. Estos resultados permiten situar la actividad de Gil y Albéniz en el contexto más amplio de la difusión de la vacunación en España, caracterizada por la interacción entre centros científicos y prácticas locales. Mientras figuras como Ignacio María Ruiz de Luzuriaga desempeñaron un papel central como articuladores de redes nacionales de difusión de la vacuna, Gil y Albéniz puede considerarse un agente intermediario a escala local, responsable de la implementación, adaptación y continuidad de la práctica vacunal en su entorno. Esta perspectiva permite entender la difusión de la vacunación no como un proceso exclusivamente institucional, sino como el resultado de dinámicas multinivel en las que los médicos locales tuvieron una participación relevante.

**Figura 1:**
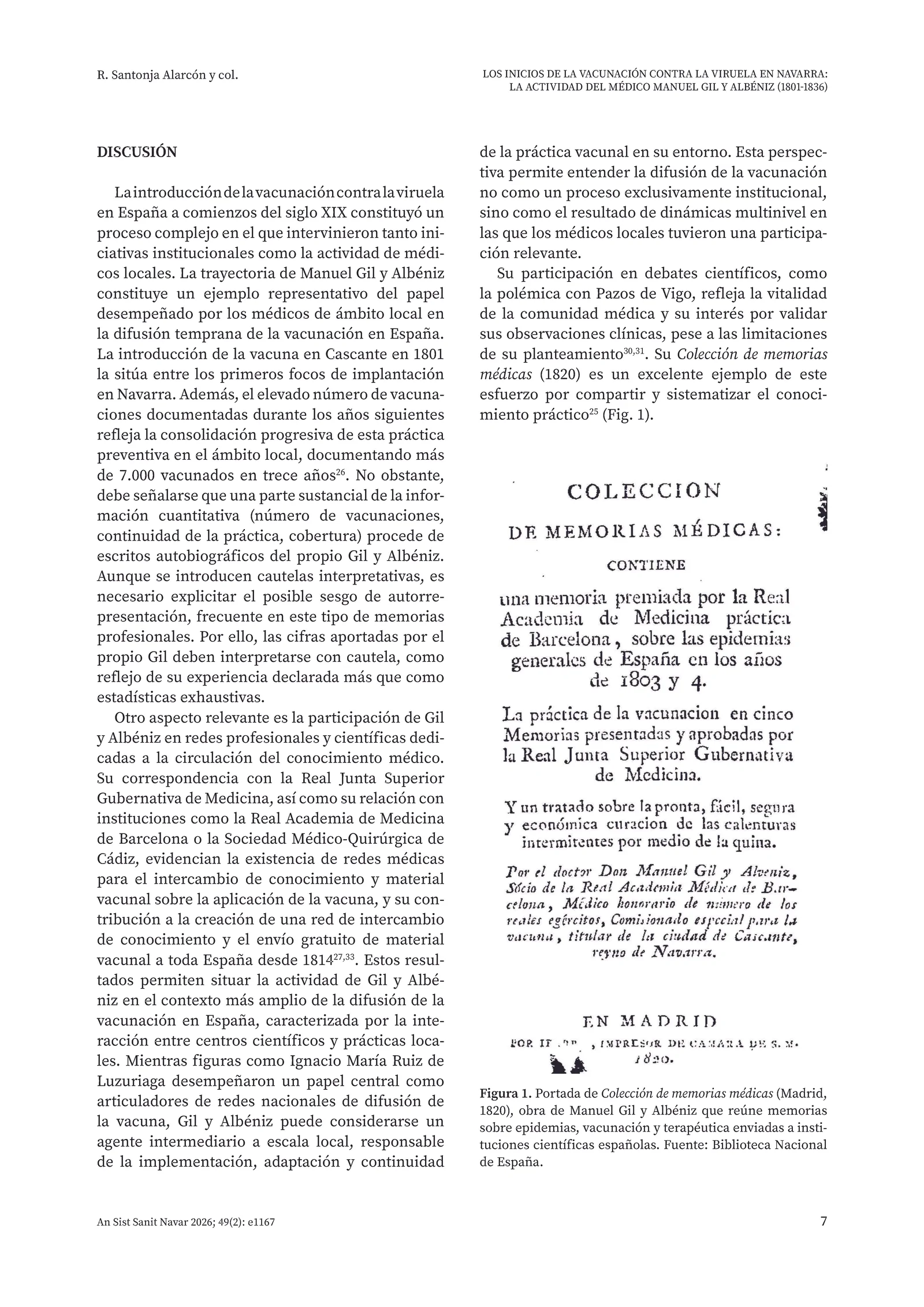
Portada de *Colección de memorias médicas* (Madrid, 1820), obra de Manuel Gil y Albéniz que reúne memorias sobre epidemias, vacunación y terapéutica enviadas a instituciones científicas españolas. Fuente: Biblioteca Nacional de España.

Su participación en debates científicos, como la polémica con Pazos de Vigo, refleja la vitalidad de la comunidad médica y su interés por validar sus observaciones clínicas, pese a las limitaciones de su planteamiento^30^^,^^31^. Su *Colección de memorias médicas* (1820) es un excelente ejemplo de este esfuerzo por compartir y sistematizar el conocimiento práctico^25^ (Figura 1).

Su biografía refleja también la compleja interrelación entre ciencia, política y sociedad. Su relación con Palafox le proporcionó apoyo y protección^36^, pero sus aspiraciones a cargos superiores (Protomedicato, pensión vitalicia) fracasaron, lo que refleja las limitaciones de su proyección institucional^15^. Su posicionamiento político como absolutista no parece haber afectado negativamente a su carrera en Cascante, lo que sugiere que, en ocasiones, las lealtades políticas no condicionaban directamente el reconocimiento profesional a nivel local. Su trayectoria representa el perfil del médico ilustrado y su contribución a la implantación de la salud pública en la España contemporánea.

Para concluir, el análisis en profundidad de la trayectoria profesional de Manuel Gil y Albéniz permite situarlo entre los introductores tempranos de la vacunación contra la viruela en Navarra a comienzos del siglo XIX. La documentación analizada muestra que la vacunación se implantó en Cascante desde 1801 y que, en los años posteriores, el propio Gil y Albéniz desarrolló una intensa actividad como vacunador y divulgador de esta práctica preventiva, alcanzando un elevado número de vacunaciones en el ámbito regional.

Además de su actividad clínica, su participación en redes profesionales y científicas contribuyó a la temprana consolidación de la vacunación en Navarra y a la difusión de una de las primeras prácticas preventivas de la medicina moderna. El caso de Gil y Albéniz ilustra el papel desempeñado por los médicos de ámbito local en la implantación temprana de innovaciones sanitarias que pueden considerarse antecedentes de la salud pública moderna.

## Data Availability

Se encuentran disponibles bajo petición al autor de correspondencia.
